# First Report of Root Rot Caused by *Phytophthora bilorbang* on *Olea europaea* in Italy

**DOI:** 10.3390/plants9070826

**Published:** 2020-06-30

**Authors:** Elena Santilli, Mario Riolo, Federico La Spada, Antonella Pane, Santa Olga Cacciola

**Affiliations:** 1 Council for Agricultural Research and Agricultural Economy Analysis, Research Centre for Olive, Citrus and Tree Fruit-Rende CS (CREA-OFA), 87036 Rende, Italy; elena.santilli@crea.gov.it (E.S.); mario.riolo@unirc.it (M.R.); 2 Department of Agriculture, Food and Environment, University of Catania, 95123 Catania, Italy; federicolaspada@yahoo.it; 3 Department of Agricultural Science, Mediterranean University of Reggio Calabria, 89122 Reggio Calabria, Italy

**Keywords:** root rot, *Phytophthora*, olive, leaf chlorosis, defoliation, wilt, molecular identification, morphological identification, ITS region

## Abstract

Leaf chlorosis, severe defoliation and wilt associated with root rot were observed on mature olive trees cv. Nera di Gonnos in an experimental orchard at Mirto Crosia (Calabria, southern Italy). An oomycete was consistently isolated from rotten roots of symptomatic olive trees. It was identified as *Phytophthora bilorbang* by morphological characters and sequencing of Internal Transcribed Spacer (ITS) regions of ribosomal DNA (rDNA). Pathogenicity was verified by inoculating potted two-month-old rooted cuttings of *Olea europaea* var. Nera di Gonnos in a soil infestation trial. *P. bilorbang* was re-isolated from roots of symptomatic, artificially inoculated olive cuttings to fulfill Koch’s postulates. This is the first report of *P. bilorbang* on *O. europaea* L. and on a species of the Oleaceae family worldwide.

## 1. Introduction

Olive (*Olea europaea L.*, family Oleaceae) originated in the Near East and spread westward in the Mediterranean basin where it is widely cultivated as a fruit tree [1,2]. Based on data from the International Olive Oil Council (IOOC), more than 10 million hectares are cultivated with olive globally and 95% of them are in the Mediterranean basin [3]. With ca. 1.1 million ha of olive groves, Italy is the second largest olive growing country in the world, with Apulia, Calabria, and Sicily regions of southern Italy accounting for about 70% of the production [4]. As a typical Mediterranean plant, olive has been traditionally cultivated in arid lands. However, during the last decades in many olive growing countries, including Italy, the olive cultivation has been extended to different types of soil and irrigation has become a common practice in olive orchards. An emerging phytopathological problem of olive trees growing in wet or waterlogged soils is root rot caused by *Phytophthora* spp. [5]. The genus *Phytophthora* (Pythiaceae, Peronosporales, Oomycota, and Chromista) comprises more than 180 described taxa [6]. With the advent of DNA sequencing the systematics of the genus evolved from morphological criteria to molecular phylogeny. Therefore, the species of *Phytophthora* were identified using molecular markers and grouped into 12 phylogenetic clades some of which have subclades [7,8,9,10]. Several *Phytophthora* species of different phylogenetic clades have been reported as causative agents of leaf chlorosis, wilting, defoliation, and twig dieback, as a consequence of root rot and basal stem cankers on olive worldwide, including *Phytophthora acerina, Phytophthora cactorum, Phytophthora cinnamomi, Phytophthora citricola sensu lato, Phytophthora cryptogea, Phytophthora drechsleri, Phytophthora inundata, Phytophthora megasperma*, *Phytophthora nicotianae, Phytophthora oleae, Phytophthora palmivora, Phytophthora pini,* and *Phytophthora plurivora* [5,11,12,13,14,15,16,17,18,19,20,21]. These species differ in aggressiveness, temperature requirements for growth, geographical distribution, and ecology. *Phytophthora palmivora*, alone or in association with *Verticillium dahliae*, was reported as causal agent of rot of fine roots and wilt of young olive trees in nurseries and new plantings in Italy [12,22]. *Phytophthora oleae* was recovered from soil and roots of wild olive trees in protected natural areas in Spain and Sicily (southern Italy) and is widespread in soil of commercial olive orchards in Calabria (southern Italy) [20,23,24]. *Phytophthora inundata* is responsible for root rot and wilt of olive trees in clay soils after flooding, acting as an opportunistic albeit aggressive root pathogen [15,18,25]. In moist environments, some soil-inhabiting species, like *P. nicotianae* and *P. oleae* have occasionally adapted to an aerial lifestyle and may infect aboveground parts of olive trees such as drupes, leaves, and twigs causing fruit rot, leaf drying, filloptosis, and twig dieback [26,27,28].

**Figure 1:**
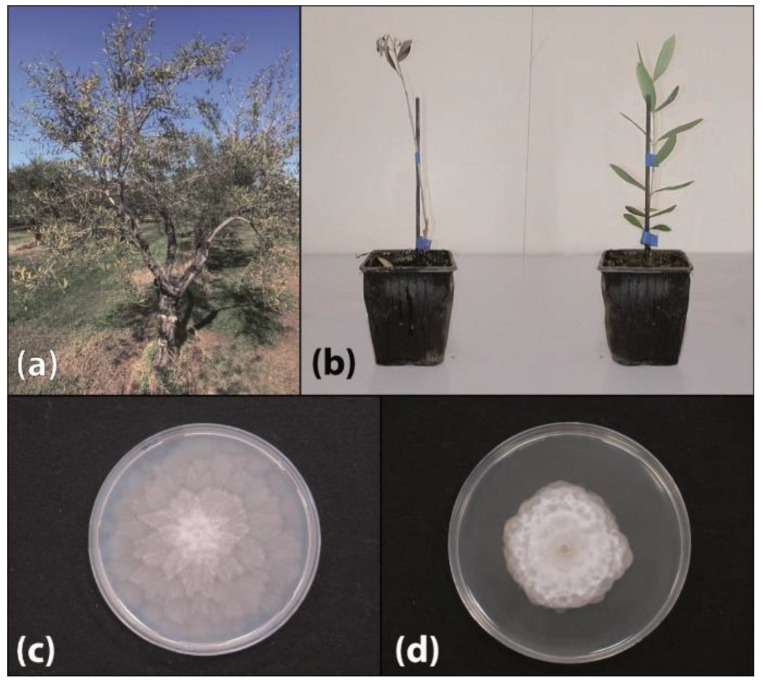
(**a**) Decline symptoms on a tree of olive (*Olea europaea*) cv. Nera di Gonnos incited by *Phytophthora bilorbang* in Calabria. (**b**) Wilt of a potted rooted cutting of olive cv. Nera di Gonnos (on the left) artificially inoculated with *P. bilorbang* through the soil, 6 weeks after transplanting into infested soil, and control non-inoculated cutting (on the right). (**c**) Morphology of 6-day-old colonies of *P. bilorbang* grown on V8 juice-agar and (**d**) on potato-dextrose-agar at 25 °C in the dark.

In autumn 2019, symptoms of defoliation, wilt, and root rot were observed on 15-year-old olive trees in an experimental orchard at Mirto Crosia, in Calabria (Figure 1a). Trees were watered in summer using a drip irrigation system. About 40% of the trees of the cv. Nera di Gonnos, originating from Sardinia (southern Italy), were affected. Symptoms were indicative of Phytophthora root rot (PRR). The main aim of the present study was to identify the causative agent of this disease.

## 2. Results

### 2.1. Species Identification and Morphological Features of Isolates

Isolations from rotten roots of symptomatic olive trees sampled in the experimental orchard of Mirto Crosia found consistently a homothallic *Phytophthora* taxon with a notable colony morphology (Figure 1c,d). Eighteen single-hypha isolates of this *Phytophthora* taxon, obtained from three independent trees (six from each tree), were characterized. They formed stellate to petaloid colonies on V8 juice-agar (V8A) and dense-felty, chrysanthemum-like and dome shaped in the center colonies on potato-dextrose-agar (PDA). Extreme temperatures for growth were 4 (minimum) and 32 °C (maximum), with an optimum at 25 °C. Sporangia formed on V8A were persistent, non-papillate, limoniform to ellipsoid and internally proliferating. Their dimensions were 45.0 to 55.0 × 25.2 to 30.2 μm, with a mean length to breadth ratio of 1.8. Globose oogonia (diameter ranging from 27.3 to 32.0 μm), paragynous antheridia and plerotic oospores (diameter ranging from 28.2 to 35.3 μm) with a thick wall (2.5 to 3.0 μm) were also observed in single cultures on V8A.

### 2.2. Molecular Identification

**Figure 2:**
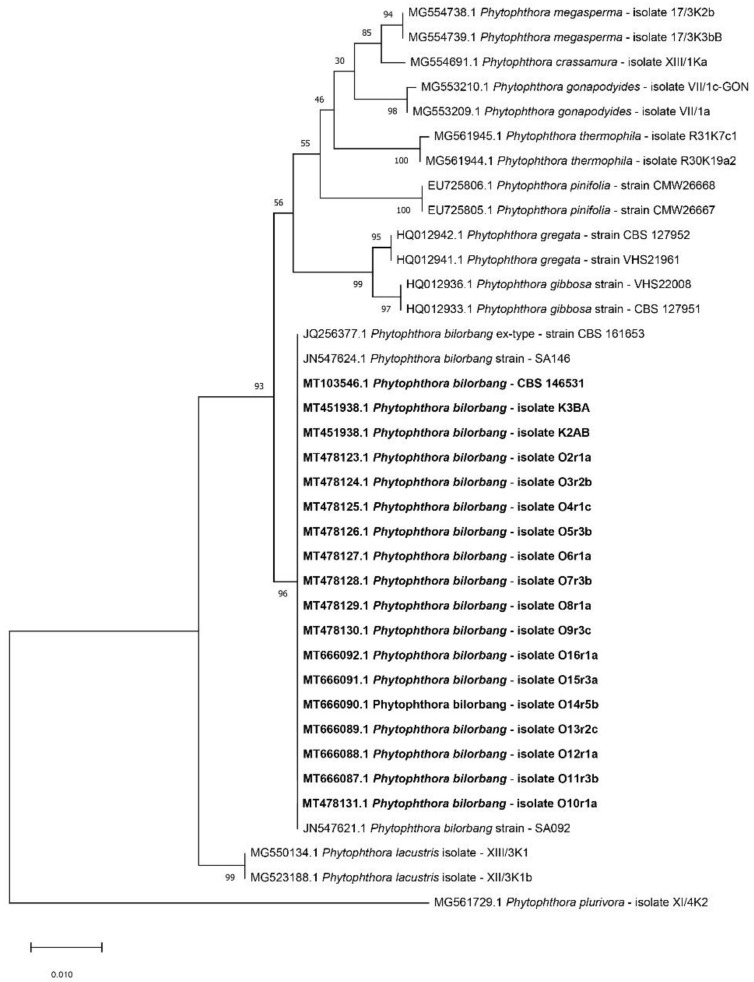
Phylogenetic tree for the ITS loci obtained by the maximum likelihood method, based on the Tamura–Nei model. Relationships between the 18 *Phytophthora bilorbang* isolates from olive (in bold), the ex-type isolate of *P. bilorbang* from European raspberry and other *Phytophthora* species within the ITS Clade 6. *P. plurivora* was used as outgroup taxon. The bootstrap consensus tree inferred from 1000 replicates is taken to represent the evolutionary history of the taxa analyzed. Branches corresponding to partitions reproduced in less than 50% bootstrap replicates are collapsed. The percentage of replicate trees in which the associated taxa clustered together in the bootstrap test are shown next to the branches. The tree is drawn to scale, with branch lengths measured in the number of substitutions per site (below the branches).

Amplification and sequencing of Internal Transcribed Spacer (ITS) regions of ribosomal DNA (rDNA) of all 18 isolates revealed 100% identity with the sequence of *Phytophthora bilorbang* ex-type CBS 161653 (GenBank Accession Number JQ256377 [29]). The phylogenetic analysis of the combined data set of sequences from ITS region of 10 out of 18 isolates recovered from olive at Mirto Crosia along with reference sequences of *Phytophthora* species within the ITS clade 6 produced a phylogenetic tree with a similar topology and high concordance with the one reported in the original description of *P. bilorbang* [29]. All isolates from olive clustered (bootstrap values of 1000 replicate) with the ex-type isolate of this species (Figure 2). The ITS sequences of all isolates from olive were deposited in GenBank (the respective GenBank accession numbers are given in Figure 2).

### 2.3. Pathogenicity

The *P. bilorbang* isolate CBS146531 from olive proved to be pathogenic on olive cuttings cv. Nera di Gonnos. All 10 rooted cuttings transplanted into pots filled with infested soil developed severe symptoms of root rot, leaf chlorosis, defoliation, wilt, and final death of the whole cutting within six weeks after the transplant (Figure 1b). Mean severity of symptoms (±S.D.) in inoculated cuttings as evaluated according to Engelbrecht et al. [30] was 4.3 ± 1.16. Conversely, control cuttings grown in non-infested potting mixture showed no aerial symptoms and no necrotic fine roots. Difference between mean severity of symptoms of inoculated and control cuttings was significant for *p* = 0.01. *Phytophthora bilorbang* was re-isolated only from roots of symptomatic cuttings; thus, fulfilling Koch’s postulates. The identity of isolates obtained from necrotic roots of symptomatic, artificially inoculated cuttings, was determined by the colony morphology, microscopy observations, and ITS rDNA sequencing.

## 3. Discussion

*Phytophthora bilorbang* was described in 2012 in Western Australia as a new species in ITS clade 6 sub-clade II, and as a pathogen of European raspberry (*Rubus anglocandicans*) [29]. Its role as the main causative agent of “raspberry decline” syndrome in Australia was further confirmed in a later study [31]. The ITS sequences of *P. bilorbang* are identical to the corresponding sequences of several isolates deposited as *Phytophthora* taxon oaksoil and an isolate deposited as *Phytophthora* taxon riversoil, whose provisional names refer to their origin, i.e., soil of oak forests and riparian ecosystems, respectively [29,32,33]. The relationship between *P. bilorbang*, *Phytophthora* taxon oaksoil, and *Phytophthora* taxon riversoil is still debated [32]. However, as the most distinctive character separating *P. bilorbang* from the other two taxa is homothallism [32], the isolates obtained from olive in Calabria were confidently referred to this species. In general, *Phytophthora* species of clade 6 have been found in forests and riparian ecosystems and, with some exceptions such as *Phytophthora asparagi*, *Phytophthora crassamura*, *P. megasperma* and, *Phytophthora rosacearum*, showing only a limited association with agriculture [34,35,36,37,38,39,40,41,42]. Additionally, *Phytophthora* species of clade 6 are predominantly sterile or homothallic in culture and appear functionally adapted to survive and thrive in aquatic environments and inundated soils [35,38]. The function of most of these species within the ecosystems is not yet fully understood. It has been hypothesized they have a prevalently saprotrophic lifestyle and their common presence and even dominance in environmental water surveys have been assumed as evidence for this hypothesis [36]. However, some members of ITS Clade 6, such as *Phytophthora pinifolia*, *P. inundata*, *Phytophthora* taxon Pgchlamydo, and *Phytophthora gonapodyides*, can be opportunistic and sometimes aggressive tree pathogens [36,37,38,39,40]. Results of pathogenicity tests on olive cuttings confirm that *P. bilorbang*, which has a prevalently aquatic lifestyle and is frequently recovered from streams and irrigation reservoirs, can be included in this group of opportunistic aggressive pathogens. It is hypothesized that in the experimental plot of Mirto Crosia soil waterlogging and asphyxiation as a consequence of flooding events or excessive irrigation predisposed the olive trees to *P. bilorbang* infections. In an extensive survey of European nurseries, *P. bilorbang* was found in rhizosphere soil of potted plants suggesting this species, like other soil-inhabitant *Phytophthora* species, can be spread worldwide through the trade of nursery plants [43].

*Phytophthora bilorbang*, like most other soil-borne *Phytophthora* species, is a polyphagous pathogen whose host-range comprises, besides *Rubus anglocandicans* (Rosaceae), plants of different families including *Alnus glutinosa* (Betulaceae), *Juniperus phoenicea* (Cupressaceae), and *Pistacia lentiscus* (Anacardiaceae) [29,40,44]. In Italy, it was recovered from rhizosphere soil and plants of the Mediterranean maquis in Sardinia (southern Italy) [40,44]. However, to the best of our knowledge, this is the first report of *P. bilorbang* as a pathogen of olive and other plants in the Oleaceae family worldwide.

## 4. Materials and Methods

### 4.1. Isolation and Morphological Identification of Isolates

In November 2019 necrotic fine roots were sampled from three distinct symptomatic olive trees cv. Nera di Gonnos in the experimental orchard at Mirto Crosia characterized by a soil with silty loam texture (Geographic Coordinates (DATUM WGS 84) 39°61′59.0″ N, 16°76′11.4″ E, Cosenza, Calabria, southern Italy). Roots were thoroughly washed in tap water, superficially disinfected in 1% NaClO for 2 min, then immersed in 70% EtOH for 30 s, rinsed in sterile distilled water, dipped dry, and plated on selective PARPNH V8-agar [45]. After an incubation period of 24–48 h in the dark at 25° C, pure cultures were obtained by transferring outgrowing single hyphae onto V8-juice agar (V8A) [11]. Purified cultures were finally obtained by single hyphal culture on V8-agar. Colony morphology and morphological features of isolates, including the morphology and dimensions of reproductive structures, were determined on colonies grown on V8A at 20–22 °C in the dark according with standard procedures [11]. Sporangia production was stimulated following the method described by Jung et al. [46]. Small fragments (size 2 mm) were cut from the growing edge of 5 to 7-d-old cultures grown in Petri dishes (15 mm diam.) on V8A at 20 °C in the dark, they were placed in a 5 cm diameter Petri dish and flooded with non-sterile soil extract water (200 g soil suspended in 1 L of de-ionized water for 24 h at room temperature and then filtered). After incubation at 20 °C in the dark for 24–72 h, dimensions, and morphological features of 50 mature sporangia of each isolate were determined at ×400 magnification.

### 4.2. Molecular Identification of Isolates

Species were molecularly identified by the amplification and analysis of Internal Transcribed Spacer (ITS) of ribosomal DNA (rDNA). To this aim, total DNA was extracted from 7-d-old cultures grown on V8-agar at 20 °C by using the PowerPlant^®^ Pro DNA isolation Kit (MO BIO Laboratories, Inc., Carlsbad, CA, USA), following the manufacturer’s instructions. The PCR amplification was performed by using the primer pairs ITS6 (5′-GAAGGTGAAGTCGTAACAAGG-3′) [47] and ITS4 (5′- TCCTCCGCTTATTGATATGC-3′) [48] in a 25 µL reaction mix containing PCR Buffer (1X), dNTP mix (0.2 mM), MgCl2 (1.5 mM), forward and reverse primers (0.5 µM each), Taq DNA Polymerase (1 U), and 100 ng of DNA. The thermocycler conditions were as follows: 94 °C for 3 min; followed by 35 cycles of 94 °C for 30 s, 55 °C for 30 s, and 72 °C for 30 s; and then 72 °C for 10 min.

Amplicons were detected in 1% agarose gel and sequenced in both directions by an external service (Macrogen, Amsterdam, The Netherlands). All the sequences were analyzed by using FinchTV v.1.4.0 [49]. Species identification was performed by blast searches in GenBank [50] and in a local database containing sequences of ex-type or key isolates from published studies. Isolates were assigned to a species when the respective consensus sequence was between 99 and 100% identical to that of a reference isolate.

Validated sequences representative of *Phytophthora* species identified within the ITS clade 6 were phylogenetically analyzed. Sequences from ex-type or authentic culture were included in the analysis as a reference [29]. Phylogenetic analysis was conducted for the ITS sequences by the maximum likelihood method, based on the Tamura–Nei model (the software MEGA-X was used).

### 4.3. Pathogenicity Test

Pathogenicity of *P. bilorbang*, isolated from roots of *O. europaea*, was tested using a soil infestation method according to Jung et al. (2017) [7]. The isolate CBS 146531 (GenBank accession number MT103546), sourced from symptomatic olive trees cv. Nera di Gonnos in an experimental orchard at Mirto Crosia, was used in pathogenicity tests. Ten potted 2-month-old rooted cuttings of olive cv. Nera di Gonnos were transplanted into free-draining pots containing a mixture of autoclaved universal potting soil (©Cifo Srl, Giorgio di Piano, Bologna, Italy) and inoculum (20 cm^3^ of inoculum per 1000 cm^3^ of potting mixture). Inoculum consisted of a 21-day-old culture of the isolate CBS 146531 grown in the dark at 25 °C in a 750 mL jar containing a sterilized medium made of 50 mL of wheat seeds and 50 mL V8-juice broth. Ten control plants were transplanted in free-draining pots containing non-infested potting mixture. After transplanting, all plants were maintained in saturated soil for 48 h and then transferred to a growth chamber at 23 °C, 80% relative humidity, and a photoperiod of 16 h of light and 8 h of dark. The trial was considered concluded when inoculated plants showed severe symptoms of decay (6 weeks post inoculation). At the end of the test, *P. bilorbang* was re-isolated from necrotic roots using the selective PARPNH V8-agar and sequenced.

Symptoms were assessed visually in accordance with Engelbrecht et al. [30]. The wilting categories reported were (1) normal (no signs of wilting or drought stress), (2) slightly wilted (slight leaf angle changes but no folding, rolling, or changes in leaf surface structure), (3) wilted (strong leaf angle change but no cell death), (4) severely wilted (very strong change of leaf angle or protrusion of veins on the leaf surface with beginning necrosis), (5) nearly dead (most leaves necrotic, some young leaves still green near the midrib, leaf angles mostly near 0°), and (6) dead (all above-ground parts dead). Data from pathogenicity test were analyzed by a two sample t-test performed by using the software R for *p* = 0.01 [51].

## 5. Conclusions

The report of *P. bilorbang* on olive in southern Italy widens the range of *Phytophthora* species involved in PRR of this typical Mediterranean crop. PRR may be a concern in recently established olive orchards and it would be useful to understand the factors fostering its worldwide and local emergence. Probably, they include the involvement of an aggressive pathogen species as well as local environmental and agro-ecological conditions, as in the case of anthracnose, a better-known emergent disease of olive [52,53,54]. Other factors fostering the emergence of PRR of olive may include climate change effects, the host range expansion of endemic polyphagous *Phytophthora* species, or the global spread of *Phytophthora* and the introduction of exotic species through the nursery plant trade [43,55].
